# 3D U-Net Segmentation Improves Root System Reconstruction from 3D MRI Images in Automated and Manual Virtual Reality Work Flows

**DOI:** 10.34133/plantphenomics.0076

**Published:** 2023-07-28

**Authors:** Tobias Selzner, Jannis Horn, Magdalena Landl, Andreas Pohlmeier, Dirk Helmrich, Katrin Huber, Jan Vanderborght, Harry Vereecken, Sven Behnke, Andrea Schnepf

**Affiliations:** ^1^ Forschungszentrum Juelich GmbH, Agrosphere (IBG-3), Juelich, Germany.; ^2^ Autonomous Intelligence Systems Group, University of Bonn, Bonn, Germany.; ^3^ Forschungszentrum Juelich GmbH, Juelich Supercomputing Center, Juelich, Germany.

## Abstract

Magnetic resonance imaging (MRI) is used to image root systems grown in opaque soil. However, reconstruction of root system architecture (RSA) from 3-dimensional (3D) MRI images is challenging. Low resolution and poor contrast-to-noise ratios (CNRs) hinder automated reconstruction. Hence, manual reconstruction is still widely used. Here, we evaluate a novel 2-step work flow for automated RSA reconstruction. In the first step, a 3D U-Net segments MRI images into root and soil in super-resolution. In the second step, an automated tracing algorithm reconstructs the root systems from the segmented images. We evaluated the merits of both steps for an MRI dataset of 8 lupine root systems, by comparing the automated reconstructions to manual reconstructions of unaltered and segmented MRI images derived with a novel virtual reality system. We found that the U-Net segmentation offers profound benefits in manual reconstruction: reconstruction speed was doubled (+97%) for images with low CNR and increased by 27% for images with high CNR. Reconstructed root lengths were increased by 20% and 3%, respectively. Therefore, we propose to use U-Net segmentation as a principal image preprocessing step in manual work flows. The root length derived by the tracing algorithm was lower than in both manual reconstruction methods, but segmentation allowed automated processing of otherwise not readily usable MRI images. Nonetheless, model-based functional root traits revealed similar hydraulic behavior of automated and manual reconstructions. Future studies will aim to establish a hybrid work flow that utilizes automated reconstructions as scaffolds that can be manually corrected.

## Introduction

The projected increases in frequency and severity of extreme weather events, combined with the expected growth of the global population, pose a risk to food security [1,2]. There is an urgent need for crop varieties and agricultural management practices that allow yield increases with sustainable use of natural resources, while being resilient to adverse growing conditions such as drought [3,4]. For optimization of plant health and yield formation, roots are of utmost importance as they determine the sites in soil where roots take up water and solutes. Root system architecture (RSA) is often a highly plastic trait that is defined by the soil conditions surrounding the root system [5].

Identifying RSA phenotypes that will perform well under adverse growing conditions is key to define breeding goals for varieties that can meet future challenges. A major constraint of identifying suitable RSAs is root phenotyping [6]. Roots can be readily observed using nondestructive techniques when seedlings are grown in rhizotrons, transparent growth pouches, or artificial growth media, but these methods usually result in 2-dimensional (2D) images that fail to capture 3-dimensional (3D) features of the root system. In addition, observations made in soil-free experimental setups are not necessarily transferable to field conditions, due to lack of root–soil interactions [7]. When roots are grown in soil, the opaque nature of the medium is a challenge. Here, roots cannot be readily observed without destroying the RSA, which hampers subsequent analysis of the plant [8]. Noninvasive methods that can derive the RSA of soil-grown plants are available but need special imaging devices. Magnetic resonance imaging (MRI)—a 3D volumetric image acquisition method widely known from medical applications—has been used for imaging soil and root systems embedded in soil in the past 2 decades [9,10].

To derive RSAs from 3D images in an efficient manner, nontrivial image processing and pattern recognition are required. In recent years, rapid progress has been made in RSA extraction and soil-related research [11–13]. Two fundamental steps are needed for deriving RSAs from MRI images: the segmentation of roots from the surrounding soil environment and the subsequent reconstruction of the root system from the segmented images [14–16]. The detection of roots in MRI is based on the difference in signal decay between water in the roots and in the soil [17]. Depending on this difference in signal decay, the segmentation task may be easy or difficult to solve. For a range of soils, the contrast between roots and soil is so high that water in soil is effectively invisible [18]. Under these conditions, the MRI scans almost exclusively contain root signals. Simple segmentation operations, such as applying a single global threshold, are adequate to remove the little noise (soil signal) contained in the data. However, achieving high contrast requires measurement settings, soil substrate, and soil water content to be chosen with care [16,19]. As shown by Pflugfelder et al. [20], soil water contents above 70% of the maximum water holding capacity become problematic in sandy soils and Brown’s soil. Although the root signal itself is unaffected, large fractions of the soil water signal cannot be suppressed and severely obstruct segmentation of roots and soil due to lower contrast. Consequently, the range of soils and experimental designs that can be readily used in MRI studies is limited, making it difficult to characterize root plasticity across a wide range of soil conditions.

Another challenge when working with MRI images are gaps in the roots. These discontinuities may originate in the MRI data itself or they may be introduced during image processing. As observed by Menzel et al. [21], ferro- and paramagnetic particles present in natural soils can lead to local, spherical signal losses and general signal deterioration. Findings of Pflugfelder et al. [20] suggest that the soil texture also influences the image quality, although a strict relationship could not be derived over the full range of tested soils. The authors recommend that the suitability of soil substrates be evaluated before they are used in MRI studies, as not all aspects affecting image quality are well defined. During segmentation, additional gaps may be introduced when the contrast-to-noise ratio (CNR) of the images is low. In these cases, applying thresholds to achieve sufficient visibility of the roots will cause additional discontinuities in the root branches [15]. This is particularly problematic with thin roots, because they may not only be interrupted by gaps but can disappear completely.

To obtain a fully connected geometry, the complete RSA structure must therefore be reconstructed from the 3D images. Reconstructed RSAs can be used to compute root system phenotyping traits [22], or they can directly be used as geometries in functional-structural root architecture models [15,23–28]. However, studies usually do not include more than 3 plants for model applications, except when automated or semiautomated reconstruction algorithms, such as RooTrak [29], are available and applicable to the respective datasets (e.g., in [23,25], where 12 RSAs have been reconstructed from micro-computed tomography image time series). Automated reconstruction methods for MRI images have been developed [14–16] but are built to work with high-quality inputs. As shown by Schulz et al. [14], capabilities of automated reconstruction algorithms for MRI are severely impeded if the input has gaps, low CNR, and/or low resolution. Although the image resolution can be increased by prolonging the image acquisition time, it comes at the cost of lower CNR [30]. Hence, manual reconstruction methods in 3D virtual reality (VR) systems [15] are still widely used to process MRI images (e.g., [24]). As manual reconstruction is a time-consuming task, data throughput in MRI root analysis pipelines is severely limited. Ultimately, improvements to CNR as well as to the resolution of MRI images are needed to extend the capabilities of automated reconstruction approaches beyond the use of high-quality inputs.

Recently, artificial neural networks have become state-of-the-art to solve many computer vision tasks, including semantic image segmentation. The rise of deep learning methods in image segmentation can mainly be attributed to their excellent abilities in discovering intricate features of interest in large datasets [31]. A popular network architecture for 2D image segmentation, the U-Net, was introduced by Ronneberger et al. [32]. In comparison to other architectures, the method is able to achieve good segmentation performance with few training samples and can rely on data augmentation when available training data is sparse [32,33]. 2D U-Nets have been successfully applied to a variety of segmentation tasks, including segmenting cells in microscopy images [34], roots and soil in rhizotron images [35], solid and gaseous phases in computed tomography (CT) images of geological material [36] and segmenting pathological lungs from surrounding body tissue in CT images. While the 2D U-net showed promising results in 2D image segmentation, the method has limited abilities when applied to volumetric images. Input data is processed slice-by-slice, ignoring 3D context information. Hence, spatial information along the vertical axis is not exploited for global feature extraction [37]. This constraint can be overcome when a 3D network architecture is applied. Çiçek et al. [38] proposed the 3D U-Net, which uses 3D volumes as inputs, and demonstrated the superior segmentation performance in comparison to an equivalent 2D implementation. Since its introduction, the 3D U-Net has been widely used in the medical field. The network was successfully applied to segment kidney volumes from confocal micropscopy images [38], prostate volumes and brain lesions from 3D MRI images [39,40], and hearth volumes from 3D CT scans [41]. Zhao et al. [42] also demonstrated promising results for the segmentation of roots and soil in volumetric MRI data. In addition to their capabilities in image segmentation, neural networks have shown excellent performance in image upsampling. They can derive super-resolution outputs using transposed convolution, where the interpolation is directly learned from the input data [43–45].

In this work, we evaluate a novel 2-step work flow for automatic MRI root system reconstruction, aimed at overcoming the aforementioned challenges. In the first step, we apply a 3D U-Net developed and trained by Zhao et al. [42] to increase CNR and resolution of MRI images by performing a segmentation into roots and soil in super-resolution. This U-Net segmentation still contains gaps and small amounts of noise. In the second step, we apply the automated root reconstruction algorithm of Horn et al. [46], which has been designed to work on imperfect and noisy data. Although both steps have been successfully validated by Zhao et al. [42] and Horn et al. [46] under technical aspects, we herein test their practical suitability for RSA trait quantification and for deriving geometries to be used in functional-structural plant models. To evaluate both steps of the automated work flow separately, we compare (a) manual expert reconstructions of raw MRI images produced using our default work flow with (b) manual reconstructions performed on the segmented images from Step 1 of the automated work flow and (c) tracings produced by the fully automated 2-step work flow. Manual reconstructions are performed using a novel, state-of-the-art VR system that allows for optimal reconstruction quality. We hypothesize that using the 3D U-Net segmentation will increase the recovered root length in manual reconstructions when compared to our default work flow, which relies on manually applied global thresholds. For the fully automated reconstructions, we hypothesize that the U-Net segmentation will allow us to process imperfect data with the automated root reconstruction algorithm and derive tracings of similar quality to the manual reconstructions. To test these hypotheses, we performed an MRI experiment with lupine plants grown in 2 different soil substrates, resulting in 2 subsets of MRI scans with vastly different image quality. We evaluate the quality of the 3 reconstruction work flows for MRI by means of visual comparisons of the reconstructed geometries and by calculating characteristic root measures. As the true RSA of plants grown in opaque soil is unknown, validation is based on comparing the reconstructed root lengths to root length data derived with WinRHIZO. In addition, we calculate model-based functional root traits. In contrast to the (isolated) characteristic root measures, these root traits allow us to investigate the integrated functional behavior of the whole RSAs in root water uptake. By comparing the equivalent conductance of the root systems, as well as the mean depth of water uptake, we can assess whether systematic differences in root hydraulic architecture between tracing methods exist (i.e., due to incorrect gap closing or differences in reconstructed root radii) and whether they are critical for their functional behavior or can be neglected. This is particularly important when evaluating the quality of the automated reconstructions to the manual reconstructions and thus determining their suitability for use in functional-structural root architecture models.

## Materials and Methods

### Experimental design

The 8 MRI scans of white lupine (*Lupinus albus*) used in this work were gathered in an experiment carried out at the Forschungszentrum Juelich. In brief, we used polyvinyl chloride cylinders (height of 21 cm, inner diameter of 5.6 cm) filled with sandy loam (*n* = 4) and natural sand (*n* = 4) to cultivate lupines. In the following, the sandy loam will be referred to as “soil”, and the natural sand will be referred to as “sand”. At the beginning of the experiment, the substrate-filled cylinders were saturated from the bottom to saturation soil water contents of 0.36 cm^3^ cm^−3^ for soil and 0.38 cm^3^ cm^−3^ for sand. Plants were grown for 8 to 15 d in a laboratory at a relative humidity of approximately 45%, a temperature of approximately 25 °C and a day–night cycle of 12 h/12 h. Photosynthetic active radiation during the day was 450 ± 50 μmol m^−2^ s^−1^. The 8 experimental containers were scanned by MRI at different time points (Table 1). Subsequently, the roots were excavated and washed. They were then scanned with an Epson flatbed scanner with a resolution of 0.005 mm in horizontal and 0.01 mm in vertical direction. The scans were analyzed with WinRHIZO (Regent Instruments, Ottawa, Canada) to determine total root length. A detailed description of the experiment is available in the Supplemental Materials (Section S1.1).

**Table 1.** Properties of soil-grown *Lupinus albus* plants at time of MRI scan.

Root system #	Substrate	Plant age (d)	Water content (cm^3^ cm^−3^)
1	Sand	14	0.36
2	Sand	14	0.31
3	Sand	8	0.33
4	Sand	8	0.32
5	Soil	14	0.34
6	Soil	15	0.25
7	Soil	9	0.30
8	Soil	8	0.32

### MRI measurements

**Fig. 1.**
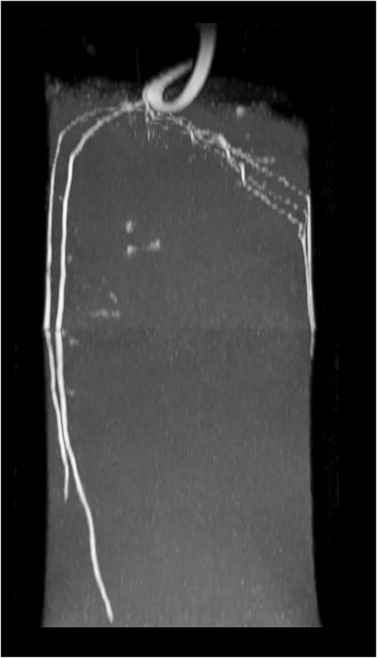
Maximum intensity projection of a MRI scan of a 14-day-old lupine root system grown in sand after dewarping and stitching (resolution 0.27 × 0.27 × 1 mm^3^).

MRI measurements were performed with a 4.7-T super-wide-bore MRI scanner (Bruker, Rheinstetten, Germany), at water contents between 0.25 and 0.36 cm^3^ cm^−3^. An overview of the parameters at scanning time is given in Table 1. Images were acquired using Bruker’s multislice multi echo imaging pulse sequence with a single echo readout (Bruker BioSpin MRI GmbH). Echo time for sand was *t_E_* = 6 ms and for soil *t_E_* = 5 ms with an acquisition bandwidth of 150 kHz, a matrix size in the horizontal plane of 256×256 points, 2 averages, and a repetition time of *t_R_* = 5 s. The axial field of view was 70 mm ×70 mm, resulting in a resolution of 0.273 mm for 70 axial slices with a thickness of 0.9 mm in interlaced mode with a gap of 0.1 mm, so that the vertical field of view was also 70 mm. Due to their large height, samples were scanned in 3 sections (top, middle, and bottom). Subsequently the 3 sections needed to be stitched together and to be dewarped due to the gradient nonlinearity artifact in our MRI system. The detailed description of the performed image processing is included in Section S1.2. An exemplary root system, resulting from the dewarping and stitching procedure, is depicted in Fig. 1.

### Root reconstruction methods

#### Two-step work flow for MRI image segmentation and root tracing

Step 1: Image segmentation in super-resolution via 3D U-Net

To improve the resolution and CNR of the MRI data, we employ the 3D U-Net previously trained and described by Zhao et al. [42]. CNR is improved by decreasing the intensity of possible noise voxels while increasing the intensity of root voxels. The U-Net increases the resolution by a factor of 2 along all axes, resulting in a factor of 8 for the number of voxels. This image preprocessing is referred to as “Step 1” of the automated reconstruction work flow.

**Fig. 2.**
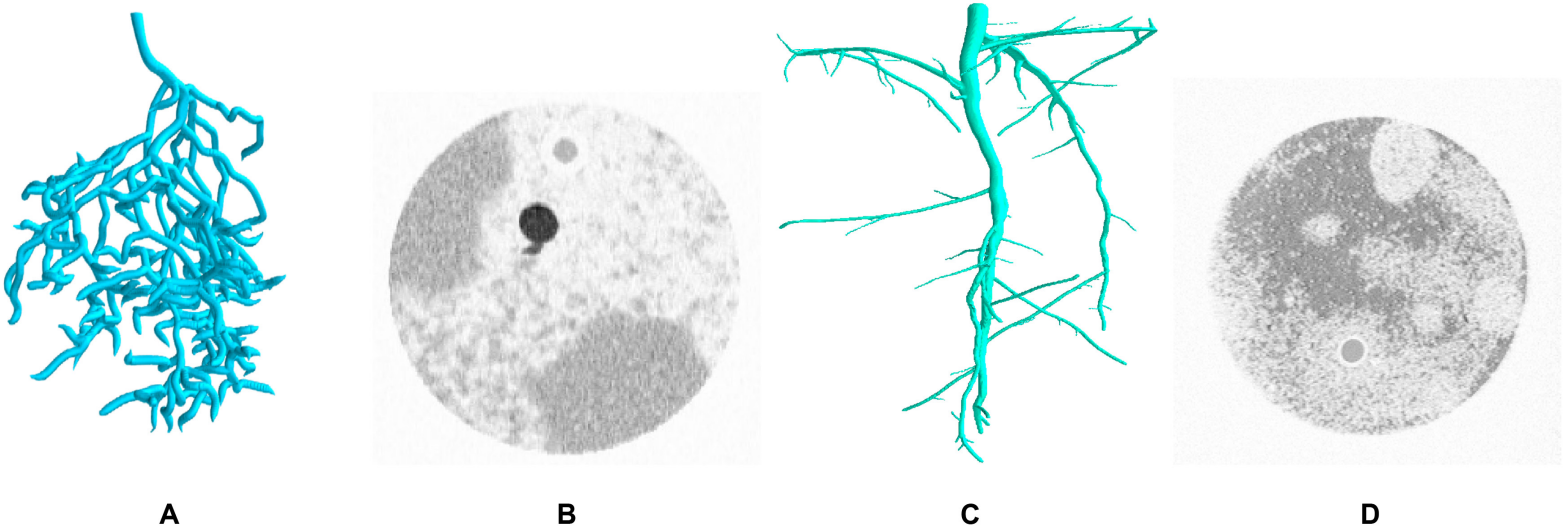
Exemplary visualization of data used for training the U-Net. (A) Rendering of manual reconstruction. (B) Virtual soil data. (C) Synthetic root system. (D) Real slice of pure MRI soil.

The dataset used to train the network was combined from 2 subsets. The first dataset (Fig. 2A and B) was generated based on 3 MRI scans of soil-grown plants and their corresponding manual root system reconstructions. The manual reconstructions (Fig. 2A) were transformed into 3D paths using thin-spline interpolation. Given a path, a 3D tube is constructed around it depending on the reconstructed radius. To increase data variety, the reconstructed radius is scaled by $\frac{1}{3}$, $\frac{2}{3}$, 1, and $\frac{4}{3}$. In a second step, these roots are rotated along the height axis by 0, 120, and 240. This results in a total of 12 different augmentations for each of the 3 manual reconstructions. To further increase variability of the training set, these augmented reconstructions were then combined with virtual soil data (Fig. 2B), simulated based on observed soil noise in real MRI images. This is combined with the second dataset (Fig. 2C and D), which consists of 30 synthetic root systems generated by randomly growing a path starting from a given shoot. After some distance, a path may split. If this is the case, a second path grows at a random angle, sampled from a defined interval, while the original path is continuous. These path are then surrounded depending on randomized radii. The resulting synthetic root systems (Fig. 2C) were then combined with noise sampled from real MRI images of pure soil (Fig. 2D). To stay within limitations of GPU memory and to allow for a deeper network architecture, the training was performed on 3D image crops of the combined dataset. Variability of the 3D image crops was again increased by augmenting image parameters, such as the contrast between root and soil. Image crops were drawn from the combined dataset and split into a training set and a validation set. Validation of the U-Net on the image crops resulted in a distant-tolerant F1 score of 0.96 [42], indicating good segmentation performance. The evaluation of the trained model, based on a test set of 5 whole (real) MRI images and their corresponding manual reconstructions, showed that it was able to detect most root branches correctly. Nevertheless, the current implementation of the U-Net does not consider connectivity of roots to the shoot during segmentation, so gaps in the roots caused by missing input information are neither recognized nor bridged. Hence, the segmented 3D images still contain false negatives corresponding to disconnections/gaps in the roots, small amounts of false positives corresponding to noise, and false positives corresponding to roots missed by the human reconstructors. Additional information on the 3D U-Net is available in the study of Zhao et al. [42].

Here, we used the 3D U-Net to increase the CNR and resolution of the MRI dataset described above. The U-Net segments the MRI images into root and soil in super-resolution. The horizontal resolution of the MRI input data is increased from 256×256 to 512×512 pixels, vertical slice distance is also halved. Subsequently, we use these segmented images in the algorithm-based reconstruction approach as well as in our manual reconstruction setup.

Step 2: Automated tracing algorithm

We use the root reconstruction algorithm developed by Horn et al. [46], herein referred to as “Step 2” of the automated reconstruction work flow, to create automated tracings (A). The algorithm itself takes a 2-stage approach and is designed to work with imperfect and noisy input data.

**Fig. 3.**
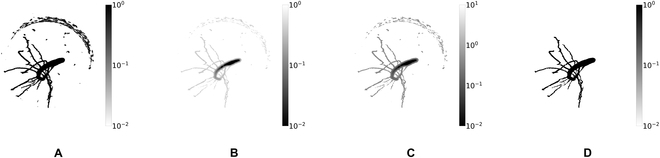
Largest-connected-component extraction with gap closing. (A) Segmented input in intensity. (B) Voxel cost. (C) Adapted cost map for gap closing. (D) Extracted largest-connected component. © [2021] IEEE. Reprinted, with permission, from Horn et al. [46].

In the first stage, the algorithm applies operations aimed at improving the input files once again, by considering the connectivity of the roots to the shoot as additional metric. Starting with the segmented input image derived by the U-Net (Fig. 3A), a start point at the uppermost shoot position of the root system is automatically set. Additionally, a minimum voxel intensity is given. Dijkstra’s shortest path algorithm [47] is used to extract the largest connected component. A cost map is derived from local radius estimates and signal intensity information and used to evaluate the cost of all voxels above the minimum voxel intensity (Fig. 3B). Low costs correspond to a high probability of a voxel being root. High-cost paths from voxels to the shoot are penalized, and paths above a defined path-cost threshold are excluded from the extraction, further reducing noise. For imperfect data (i.e., data with gaps), this rigid exclusion of low-intensity voxels and high-cost paths means that portions of the root system will not be extracted. To address this issue, the shortest path algorithm is modified and extended with an option to bridge gaps of a predefined maximum gap length. An updated cost map with enhanced contrast between gap and no-gap voxels is created to allow the algorithm to connect discontinuous root segments under the defined maximum gap length (Fig. 3C). The largest connected component resulting from this first stage is a binary volume that excludes noise clusters that are farther than the maximal gap length from the roots and in which root segments are connected by a unique connection (Fig. 3D). This fully connected volume now allows the extraction of a root skeleton.

In the second stage, a modified version of the 3D curve skeletonization algorithm described in the study of Jin et al. [48] is used to extract a root structure graph. A detailed description of the gap-closing modification to Dijkstra’s shortest path algorithm [47] and the modification to the 3D curve skeletonization algorithm by Jin et al. [48] is given in Sections S2.1 and S2.2. Further information is also available in the study of Horn et al. [46].

#### Manual tracing in VR

We developed and deployed a new VR system for the manual tracing of root systems. Unreal Engine is utilized as frontend and Python/VTK as backend for the computation of geometries from MRI scans. Geometries are visualized as opaque marching cubes isosurface and can be dynamically adjusted by applying different signal cutoffs (global thresholds) to the intensity values of the 3D images. This allows to define the desired signal contrast between roots and soil and also to change it during a reconstruction. The user wears a head-mounted display and interacts with the VR system by using tracked controllers, whose position and orientation are indicated by a digital copy in VR. The displayed data can be moved, scaled, and rotated to give the user an optimal perspective on different areas of the root systems in VR. We chose Root System Markup Language as described by Lobet et al. [49] as output format, a data format for RSA widely used in phenotyping and modeling applications. The hardware setup consists of a HTC VIVE Pro head-mounted display with HTC VIVE controllers (version 2018) connected to a midrange desktop computer with a NVIDIA GeForce RTX 2060 SUPER GPU, an Intel Core i7-8700K CPU, and 32 GB of RAM.

**Fig. 4.**
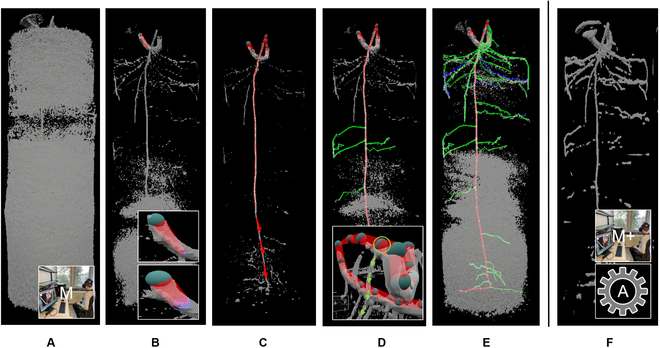
Manual root system reconstruction work flows based on images from the VR application. Shown are the marching cubes isosurfaces of an MRI scan at different signal thresholds (gray) and manual tracings with color-coded root orders at different reconstruction stages. (A) Raw image of MRI scan as displayed in VR. (B) Threshold adjustment for improved visibility of roots, drawing and adjusting the radius of the first tap root segment. (C) Reconstructing the tap root while creating nodes at potential branching points. (D) Reconstruction of laterals from bottom to top. (E) Finalized manual tracing. (F) U-Net segmentation in VR. Work flow M is based on (A), M+ and A are based on segmented images (F).

The manual reconstruction work flow in VR is displayed in Fig. 4. For this schematic illustration, the point of view in VR was kept constant. To start the root system tracing, the user loads a raw image file of a scan into the VR system (Fig. 4A). The user then picks a signal threshold that allows to differentiate roots and soil as good as possible. Next, a parenting node (i.e., the uppermost point of the tap/primary root) can be defined by clicking on the respective position on the opaque isosurface. A circular disk appears at the position of the node and can be scaled to the radial dimensions of the isosurface to define the node radius. Now, the user follows the isosurface resembling the tap/primary root and defines a second node (Fig. 4B). As soon as the tap/primary root consists of 2 segments (Fig. 4C), lateral roots can be created by selecting an inner node and drawing a new root segment (Fig. 4D). The VR system also allows corrections and manipulations of the constructed root graph.

Manual tracing was performed by a single person to avoid human reconstruction bias. First, we reconstructed the tap/primary root top to bottom. At all visible branch points, we made a tap/primary root node to facilitate later tracing of laterals (Fig. 4C). Then, laterals were reconstructed following the tap/primary root from bottom to top (Fig. 4D). We aimed at reconstructing as many roots as possible (Fig. 4E). Depending on the CNR of an image, the number of gaps, and their length, this may require applying multiple global thresholds and much manual gap closing. The manual gap closing relies on educated guesses that consider similarities in appearance, radius, orientation, position, and trajectory of disconnected root segments.

First, we used this system to perform a manual tracing based on the raw MRI images (M) (Fig. 4A). Multiple adjustments of the threshold were needed to achieve sufficient visibility of all roots. Second, we used the system to perform manual reconstructions based on the images segmented by the 3D U-Net (Fig. 4F), subsequently termed M+. Due to memory restrictions of the manual reconstruction setup, the super-resolution outputs from the U-Net needed to be scaled down. To achieve this, the U-Net was adapted to map the super-resolution outputs to the original scan resolution of 256 × 256 pixels. The initial threshold of our manual reconstruction setup (30% of maximum signal-intensity) provided a good balance of roots and noise. Hence, no adaption of this threshold was needed when working on the segmented images.

### Measures to determine success

#### Visual comparison of tracings

**Fig. 5.**
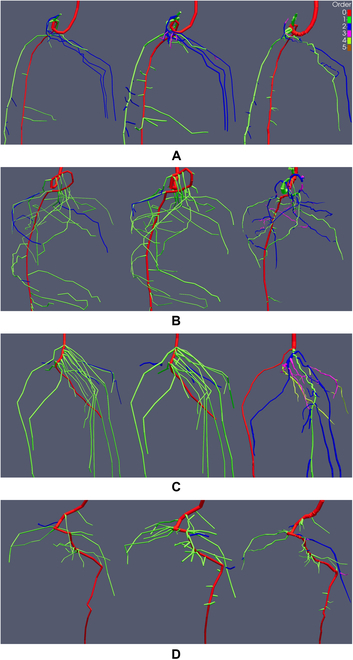
Manual tracings M (left), manual tracings after segmentation M+ (middle), and automated tracings A (right) of 4 Lupinus albus root systems (A to D) grown in sand derived by MRI scans. Reconstructions are cropped to show areas of interest. Colors display root orders, and root segments are scaled by their respective radius. Age of the root systems is between 8 and 14 d (see Table 1).

**Fig. 6.**
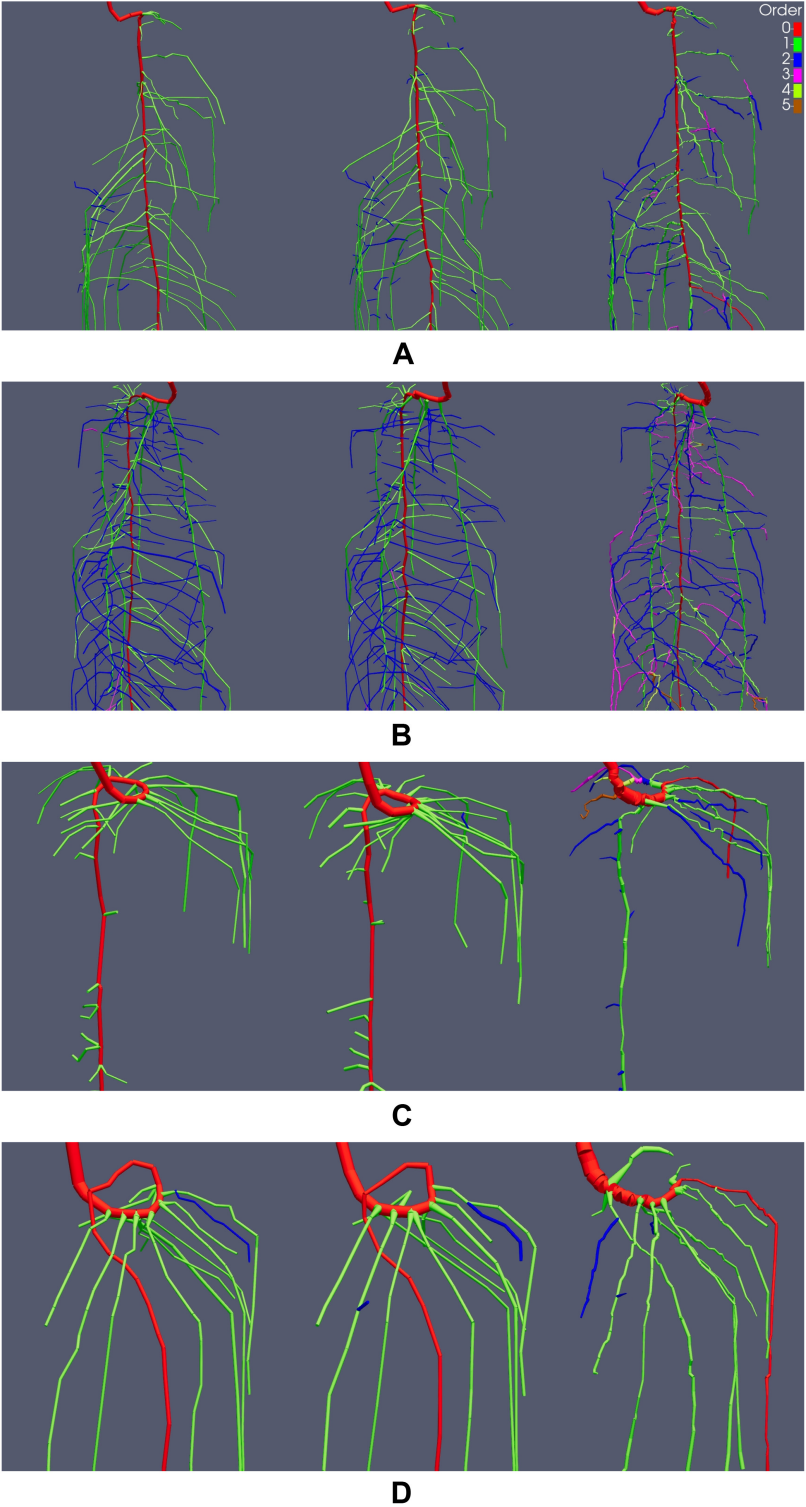
Manual tracings M (left), manual tracings after segmentation M+ (middle), and automated tracings A (right) of 4 Lupinus albus root systems (A to D) grown in soil derived by MRI scans. Reconstructions are cropped to show areas of interest. Colors display root orders, and root segments are scaled by their respective radius. Age of the root systems is between 8 and 15 d (see Table 1).

We show the RSAs resulting from the M, M+, and A reconstruction methods of MRI images, with the root order per segment color-coded (Figs. 5 and 6). RSAs in the result section are cropped to highlight areas of interest. The complete reconstructions are accessible in Figs. S3 and S4. Root segments are scaled with the radius determined by the respective reconstruction method. The visualization allows qualitative evaluation of differences between the manual reconstruction methods, due to working on raw MRI data or on the U-Net segmentation (Step 1, see Fig. 4A and F), as well as evaluation of the volume extraction and topological tracing performed by the algorithm in the A tracing.

#### Quantitative measures

We calculate a selection of common root measures [50] to describe RSA and robustness against reconstruction errors of the root systems obtained by the M, M+, and A reconstruction methods. In the results section, the metrics of each reconstruction method are aggregated over the root systems and presented in tabular form. An overview of the calculated root system measures is given in Table 2. We use CPlantBox [51,52] to load the Root System Markup Language files of the tracings and then calculate the root measures. In addition to the common root measures, we evaluate differences in root hydraulic architecture of the reconstructions by calculating the model-based functional root system metrics *K_rsc_* and zSUF for 2 scenarios. In the first scenario, called constant scenario, we calculate the functional metrics by applying the same fixed axial and radial conductivities to all roots. For the second scenario, called variable scenario, we apply order and age-dependent root hydraulic properties. Used age-distribution and conductivity values are accessible in Figs. S1 and S2.

**Table 2.** Characteristic root system measures.

Variable	Description
RL*_WR_*	Root length measured by WinRHIZO (cm)
CNR	Contrast-to-noise ratio (-)
RL	Root length of reconstruction (cm)
Recovery rate	Recovered root length against WinRHIZO (%)
RLD	Root length density (cm cm^−3^)
HMD	Half-mean distance (cm)
*r_mean_*	Mean radius of root system (cm)
*K_rsc_*	Equivalent root system conductance for constant root hydraulic properties (cm^2^ d^−1^)
*K_rsv_*	Equivalent root system conductance for variable root hydraulic properties (cm^2^ d^−1^)
zSUF*_c_*	Standard uptake fraction for constant root hydraulic properties (cm)
zSUF*_v_*	Standard uptake fraction for variable root hydraulic properties (cm)
*vr*	Manual reconstruction speed (cm min^−1^)

Three additional measures, not directly related to the RSAs, are included in the results section to enable a more in-depth classification of the results. The available root length information from WinRHIZO measurements, RL*_WR_*, is used to calculate the recovery rate. The recovery rate is the best available metric for quantitative validation of root system reconstructions of plants grown in opaque soil. For the M and M+ reconstructions, we also report the respective reconstruction speed, *vr*. To give an estimate of the quality of the MRI images, we calculate an exemplary CNR for the raw images. All equations used for the calculation of the variables in Table 2 are given in Section S3.

#### Statistical analysis

All quantitative measures, except CNR, were statistically analyzed using R 4.2.1 [53] with RStudio 2022.2.3.492 [54] and the packages rstatix 0.7.0 [55] and ggpubr 0.4.0 [56]. Because of systematic differences in RL and CNR of plants grown in sand and soil of the MRI experiment, we divided the 8 plants into 2 groups and performed separate statistics. As we are interested in the differences between the applied reconstruction methods and not in the differences between the root systems, we subsequently grouped the reconstructions according to the reconstruction methods M, M+, and A resulting in 3×4 reconstructions per substrate. Consequently, we consider the root systems as subjects (*n* = 4), the reconstruction methods as repeated measures of the same subject (i.e., within-subject factor with 3 levels), and the root metrics as the dependent variables. In the results section, we only report the mean values of the groups M, M+, and A. The individual values per reconstruction method and root system are included in Tables S1 and S2.

We tested the within-subject levels of each analyzed dependent variable for normal distribution by means of Shapiro-Wilk tests. With the exceptions of total root tips, second- and third-order laterals for the reconstructions of the MRI*_sand_* subgroup, and second- and third-order laterals for the reconstructions of the MRI*_soil_* subgroup, the assumption of normality was met. For the variables following normal distribution, we performed repeated-measures analysis of variance (ANOVAs) to test for significant differences between the reconstruction methods. During the ANOVA, the criterion of sphericity was tested. In case sphericity was not met, a Greenhouse–Geisser correction was applied. If the repeated-measures ANOVA revealed significant differences between the reconstruction groups, we performed multiple pairwise, paired, 2-sided *t* tests (M to M+, M to A, M+ to A), to locate significant differences between the groups. Due to the multiple comparisons, the *P* values of the ANOVA were corrected with the Holm–Bonferroni method. For the aforementioned cases of non-normally distributed variables, we performed a Friedman test as nonparametric alternative. All statistical tests were performed at *α* = 0.05. We report significant differences of the post-hoc tests in the tables displaying the root measures. Significant differences found by the repeated-measure ANOVAs are indicated by superscript lowercase letters, and significant differences found by the Friedman test are reported by superscript uppercase letters. If no letter is specified, the mean values are statistically indifferent.

## Results

### Visual comparison of tracings

RSAs of the 8 lupine root systems derived by the M, M+, and A reconstruction methods are displayed in Figs. 5 and 6. The age of the root systems is between 8 and 15 d (see Table 1). Note that the MRI*_sand_* and the MRI*_soil_* datasets each consist of 2 younger (≈8 d) and 2 older (≈15 d) plants. Therefore, we observe a large variability in appearance between the root systems of the same dataset caused by the age differences but also between the root systems of the 2 datasets obtained in different soil substrates. For the root systems grown in sand (Fig. 5), we can observe differences in root lengths between the M+ and M reconstructions. With the exception of the root system in Fig. 5C, all M+ reconstructions include roots that are not present in the M tracings. The additional roots are mainly of first order. We also observe slight increases in the length of some roots that are detected in both reconstructions, being longer in the M+ reconstruction. Except for the additional root length included in the M+ reconstruction, the similarity of roots present in both manual reconstructions in terms of root order, root orientation, and root position is very high. Therefore, working on the segmented images does not have much impact on human decision making for roots that can also be identified when working on the raw MRI images. An exception to this observation is the mean root radius, which is qualitatively larger for the M+ reconstructions.

When we compare the volumetric extraction of the A tracings to the manual tracings, we observe differences to M as well as to M+. Qualitatively, the difference in root radii between A and M+ is smaller than between M and M+. The total root length of A is lower than in M and M+ (see e.g., Fig. 5B), with A being more similar to M than to M+. Additionally, we observe an increased amount of directional changes in the root trajectories of A. These frequent changes in direction can lead to step-like root trajectories (see, e.g., upper half of Fig. 5C). Some roots present in both manual reconstructions are missing in A (see, e.g., lower section of Fig. 5B). This indicates that a portion of the gaps present in the U-Net segmentation is still too large to be successfully bridged by the algorithm. Furthermore, we can observe that parts of the root systems in A have different connectivity than in M and M+. As visible in the upper-right part of the root system in Fig. 5A, the algorithm traces the 3 second-order lateral roots differently than the human reconstructor. Here, gaps in the segmented image could be bridged to some extent. However, gaps too large to be bridged eventually result in partial root losses as well as in different connectivity of the recovered root segments. Incorrect gap closing seems to occur especially when gaps between interrupted segments of the same root are larger than the distance to an uninterrupted root in direct vicinity. The different connectivity caused by the partial recovery then shifts order of the respective segments toward higher values. Further topological errors in A may be caused by the fact that the topological tracing logic applied by the algorithm is not yet suitable for all cases. In case of root system in Fig. 5C, the algorithm identifies a wrong root as the tap root, although the extracted volume suggests other candidates. Again, the orders are shifted toward higher values when compared to M and M+.

For the 4 root systems grown in soil (Fig. 6), differences between the extracted structures are smaller than for the systems grown in sand. The M+ reconstructions do not include substantial amounts of additional roots or longer roots than the M reconstructions. In terms of the extracted volumes, same holds true when comparing M and M+ to A: although there are still some gaps present (e.g., upper third of root system in Fig. 6A), the volume extraction of the algorithm is more complete. However, the topology derived on the extracted volumes again shows errors. In cases of the root systems in Fig. 6C and D, the errors are caused by gaps in the upper part of the tap root, which could not be successfully closed by the algorithm. Additionally, we observe errors in topology that are related to merging root structures in the volume extraction. Such merged structures can be caused by roots that are in direct contact with each other. An example of this issue can be seen in the upper region of the A reconstruction in Fig. 6C. The uppermost second-order lateral root emerging from the tap root splits into 2 separate roots. When compared to the manual reconstructions, it becomes apparent that this second-order lateral root actually consists of 2 separate second-order laterals. Therefore, it should be connected to the tap root by 2 separate connections that do not branch later on. In the cases of root systems in Fig. 6A and B (see also Fig. S4B), topological errors related to the topological tracing logic of the algorithm seem to cause a wrong trajectory of the tap root in the lower third of both root systems. The same applies to other parts of the A tracings, e.g., the ring at the bottom of the system in Fig. 6B (see also Fig. S4B), where the volume extraction should generally allow a more precise determination of root orders.

### Quantitative measures

Table 3 shows the root measures derived for the MRI root systems grown in sand and soil. The CNR of the MRI images derived for the plants in the 2 substrates differs substantially. Images acquired in sand have a CNR of 11, resulting in poor contrast between roots and soil and a large number of gaps in the roots, which are also of considerable length. MRI images taken in soil have a comparably high CNR of 171, translating to much better contrast between roots and soil and less missing information in form of gaps. This is probably due to the high soil water content at scanning time, which is MRI visible in sand, other than in soil. The slower relaxation in nearly saturated sand cannot be faded out completely by the choice of the weakly *T*_2_-weighted pulse sequence with *t_E_* = 6 ms: latter is a compromise between getting sufficient signal from roots and suppressing the signal from sand. For soil with its inherently fast relaxation, the sequence works far better.

**Table 3.** Comparison of root measures for *Lupinus albus* tracings derived by MRI scans. MRI*_sand_* gives the mean values of the 4 root systems grown in sand (see Table S1), MRI*_soil_* gives the mean values of the 4 systems grown in Kaldenkirchen soil (see Table S2). M denotes manual tracings derived using unaltered MRI images, M+ denotes manual tracings performed on the U-Net segmentations, and A denotes tracings derived by the 2-step automated work flow. Superscript lowercase letters denote statistically significant differences between the mean values of the reconstruction types M, M+, and A within the dataset MRI*_sand_* and MRI*_soil_*, as determined by repeated-measures ANOVAs and located between the mean values of the groups by 2-sided *t* tests. Superscript uppercase letters indicate significant differences between the mean values as determined by Friedman tests and located between the mean values of the groups by 2-sided *t* tests. If no letter is specified, the mean values are statistically indifferent (see the statistical analysis section). Descriptions of the quantitative measures are given in the quantitative measures section, and equations of measures and descriptions of the constant and variable simulation scenarios are given in Section S3. Note that *K_rs_* and zSUF are simulated and not measured quantities (see Eqs. S4 to S6).

Dataset	MRI*_sand_*			MRI*_soil_*		
Reconstruction method	M	M+	A	M	M+	A
CNR (-)	11	-	-	171	-	-
RL (cm)	92	110	85	226	231	221
Recovery rate (%)	64*^ab^*	78*^a^*	60*^b^*	88	91	84
RLD (cm cm^−3^)	0.20	0.24	0.19	0.50	0.51	0.48
HMD (cm)	1.3	1.2	1.4	1.0	1.0	1.1
*r_mean_* (mm)	0.26*^a^*	0.34*^b^*	0.32*^ab^*	0.26	0.28	0.26
# of roots (-)	22	36	53	79	91	111
# of first laterals (-)	17	28	20	38	38	25
# of second laterals (-)	4	6	18	38	50	53
# of third laterals (-)	0	1	11	2	2	24
*K_rsc_* (cm^2^ d^−1^)	2.0E−03*^a^*	2.8E−03*^b^*	2.4E−03*^ab^*	3.3E−03	3.5E−03	3.2E−03
*K_rsv_* (cm^2^ d^−1^)	1.0E−02	1.3E−02	1.1E−02	1.3E−02	1.4E−02	1.2E−02
zSUF*_c_* (cm)	−4.5	-4.3	-4.6	−6.5	−6.3	−6.5
zSUF*_v_* (cm)	−3.5	−3.1	−3.5	−4.3	−4.1	−4.1
*vr* (cm min^−1^)	3.3^a^	65^b^	−	5.8^a^	7.4^b^	−

For the root systems grown in sand, the quantitative measures support the initial findings of the visual comparison. We see differences in RL between M, M+ and A, translating to recovery rates of 64% for M, 78% for M+, and 60% for A. Significant differences are found for the pair {M+,A} but not for the pairs {M,M+} and {M,A}. Root length density (RLD) and half-mean distance (HMD) of the reconstructions follow the same pattern as RL and are therefore on a similar level as RL for all reconstruction methods. As root growth is confined by the experimental containers, we observe a linear increase of RLD with increasing RL. The mean radius, *r_mean_*, derived in the manual reconstructions M and M+ is significantly different, with a higher radius occurring when working on the segmented images, while there are no significant differences to the mean radius determined in the A tracings. The total number of roots found by M+ is higher than M. These additional roots are almost exclusively first-order laterals.

Although the RL is lower, there are more roots found in A than in both manual reconstructions. These additional roots are of higher orders, as well as of orders (>higher than third-order lateral) that are generally not detected by the human reconstructor. Remembering the visual comparison, this inflation of root order is to be expected. It is likely associated to errors made by the algorithm and not to errors made during the manual reconstruction: partial gap closing leads to different connectivity, and combined with general problems in the topological decision making of the algorithm (e.g., incorrect determination of the tap root), the distribution of roots per order is skewed to higher values. Although these issues inflate the root orders in A, partial gap closing makes the other root measures more robust, as it still helps to recover larger fractions of RL.

Interestingly, the differences in RL and connectivity between the manual reconstructions and A do not transfer directly to the *K_rsc_* values. We observe significant differences between M and M+, while the *K_rsc_* value of the A tracings is statistically equivalent to M and M+. As this simulation scenario applies the same axial and radial conductivity values to all root segments, the water uptake of a root segment largely depends on its respective root radius. Hence, the higher similarity in mean root radii between M+ and A has greater impact on *K_rsc_* than the observed differences in root length and connectivity of A to M and A to M+. The mean depth of root water uptake, zSUF*_c_*, is statistically indifferent for all reconstruction methods. Again, values of M+ and A are more similar than M to M+ and M to A. Root water uptake in the constant scenario can be allocated to a mean depth of approximately 4.4 cm over all reconstruction methods. The more realistic parameterization of the variable simulation scenario that explicitly assigns different radial and axial conductivities to the tap root and first-order laterals, as well as varying them for all root segments based on their respective radii and interpolated age, does not result in larger differences of the root water uptake metrics. *K_rsv_* of M+ and A are further harmonized. We see that this parameterization decreases the mean depth of root water uptake to values between 3.1- and 3.5-cm depth. The difference of M+ to M as well as to A is higher. This can be explained by the fact that all root systems possess a high number of laterals in the upper region. As the variable scenario attributes a higher radial conductivity to the lateral roots, root water uptake is shifted toward areas with high RLD and zSUF*_v_* decreases. The effect is more pronounced for M+, since increases in recovery rate are mainly obtained in the upper region of the root systems (see, e.g., Fig. 5B and D).

The difference in reconstruction rate *vr*, calculated according to Eq. S8, between M and M+ is statistically significant. For the M work flow, we record an average *vr* of 3.3 cm root per minute. This rate is almost doubled (+97%) when working on the U-Net segmentation in M+. For images gathered in sand, the manual reconstruction is greatly hindered by the poor CNR. In addition to substantial gaps in the data that have to be connected to the fullest extent possible, noise prohibits identification of small and thin root segments severely. When performing the M reconstructions on unaltered images, multiple thresholds have to be set in order to identify the general trajectory of the roots, as well as to recover unconnected parts of the roots within gaps. This results in much larger reconstruction times. When working on the segmented image, in which most of the soil signal is successfully removed, the gap closing becomes less challenging. Visibility of roots is improved and closing gaps is easier because the gaps are not as numerous or as large as in the thresholded raw data.

For plants grown in soil, differences between the 3 reconstruction types are small. We record RLs of 226 cm for M, 231 cm for M+, and 221 cm for A. Resulting recovery rates range between 88% and 91%. Due to the highly similar RLs, differences in RLD and HMD are also small. In this case, we also observe higher similarity of the mean root radii for all reconstruction methods. The significant difference in mean radius between M and M+ is not present for the reconstructions of soil. The number of root tips is slightly increased from 79 to 91, when comparing M to M+, suggesting that the small increase in RL can largely be attributed to finding additional roots. More specifically, the exact same number of first-order laterals is recorded while the increase in total number of roots is solely composed of second-order laterals. Again, the segmented image allows detection of additional roots that are of the same order as present in the M reconstruction. Although the number of total roots found in A is the highest, the number of first-order laterals in A is lower than in M and M+. Once more, an increased number of ≥ third- and higher-order laterals (see Fig. 6), suggests that this increase is caused by partial gap closing combined with general errors in the topological decision making of the algorithm. *K_rs_* and zSUF of all 3 reconstruction methods are highly similar for the constant and variable simulation scenarios, indicating equivalent behavior in root water uptake. Again, we observe an increase in the reconstruction rate *vr*. Although not as prominent as for the root systems grown in sand, we still record a statistically significant increase of 27% in *vr*. The reasons for this increase are the same as for the root system grown in sand, but here, the manual thresholding achieves a more similar quality to U-Net segmentation.

## Discussion

In this work, we tested a novel 2-step work flow for automated root system reconstruction from noisy, imperfect 3D MRI images. Both steps of the automated work flow were investigated for their suitability to improve or replace the currently used manual work flows—under practical conditions. We could show that 3D U-Net segmentation provides fundamental improvements to the manual work flow for the low CNR dataset MRI*_sand_*. Substantial increases in mean reconstruction rate (+97%), in root length (+20%), and in root recovery rate (+14%) could be achieved (see Table 3). For the MRI*_soil_* dataset with a high CNR, the benefits of using the U-Net segmentation were smaller: reconstructed root length was increased by 2%, root recovery rate by 3%, and reconstruction rate by 27%. These results are consistent with our initial hypothesis. When CNR is low, manually set thresholds have limited capability in segmenting the images into root and soil, which is in line with results reported by Pflugfelder et al. [20] for data derived under similar conditions as the MRI*_sand_* dataset. It is tedious and time-consuming to achieve sufficient visibility of the whole target structure. Multiple thresholds need to be applied, since root signal intensities vary over a wide range and are close to or overlap with soil intensity values. Imposing these thresholds results in a substantial number of gaps in the root structure and loss of smaller roots, as low-intensity parts of the root system will be cut off. This cutoff of low-intensity values also led to significant differences in mean root radii of M and M+ (see MRI*_sand_* in Table 3). At low CNR, applying high signal intensity thresholds to increase root–soil contrast can thin out roots, especially low-intensity root signals at the root–soil boundary. Under these low CNR conditions, the 3D U-Net offers a segmentation performance that cannot be matched by manual thresholding. Gaps in the target structure are less frequent, smaller, and a higher number of low-intensity roots that are close to the signal intensity of the unsuppressed soil signal are still preserved in the segmented images. The additional roots found when working on the segmented images in M+ were of the same orders as present in M. Hence, the U-Net segmentation increased the general visibility of roots but did not allow the identification of potentially present thinner roots of higher orders. On average, the M+ reconstructions still lacked ≈22% of the roots in sand and ≈9% in soil. As the performed WinRHIZO analysis did not derive order-specific root measures, we cannot characterize this missing root fraction precisely. In general, MRI protocols suitable for deriving RSAs from soil-grown plants have a minimum detectable root radius, which, for example, was experimentally determined to be ≈0.1 mm for the protocol used by van Dusschoten et al. [16]. On the other hand, the increase in recovery rate of M+ in sand can be strictly attributed to finding additional roots of similar radius than found in M; same is true for the small increase in recovery rate in soil. In future experiments, the WinRHIZO analysis should include a quantification of root measures per root order. This would allow us to determine whether the missing root fraction consists of additional roots with similar radii to the detected roots or whether a fraction of the roots is below the MRI detection limit.

For images with high CNR, the benefits of the U-Net segmentation in manual reconstruction are obviously reduced. As shown for the MRI*_soil_* dataset, root metrics derived by M and M+ are highly similar (see MRI*_soil_* in Table 3). The intensity thresholds needed to suppress the soil signal are smaller than in sand. Consequently, the higher contrast allows more suitable thresholds that exclude smaller portions of the root signal. The 27% increase in reconstruction speed is nonetheless interesting, as it emphasizes an additional benefit of the U-Net segmentation: decrease of human reconstruction bias due to a more complete target structure. Most of the time spent on manual reconstruction of raw MRI images was on finding appropriate thresholds and interpreting gaps. As shown by Bauer et al. [57] for the case of 2D rhizotron images, the variability between individuals reconstructing imperfect images (i.e., gaps in the target structure) can be large. Based on our experience, this also applies to 3D MRI images. It can be assumed that the amount of missing information in the input files is strongly correlated to the divergence of reconstructions performed by different individuals. One aspect of this divergence is the general ambiguity that is introduced by gaps in the data, as gap closing is a subjective task. Small and isolated gaps require little interpretation, while a large number of gaps of considerable length leads to ambiguity in the interpretation of the target structure (see Fig. 4). Another aspect are the thresholds chosen to visualize the data. Depending on time expenditure and initial guesses, this procedure can have a certain hit-or-miss character. As the U-Net segmentation offers a way to standardize the thresholding procedure while reducing the amount of missing information in the target structure, we conclude that this image preprocessing approach should lower human reconstruction bias in manual work flows severely. Since the use of the segmented images also increases the recovery rate and the reconstruction speed, we propose that an improved manual work flow for MRI images, as demonstrated in this work with the M+ work flow, can be created by utilizing the U-Net segmentation.

Finally, the differences in recovery rates between the MRI*_sand_* and MRI*_soil_* datasets highlight the need for careful interpretation of the derived root metrics. Although we could decrease the difference between low- and high-contrast data, there still is a systematic difference in the recovered root length for the 2 substrates (i.e., ≈13% between M+ of sand and soil). Since the amount of roots obtained from MRI data has been shown to vary depending on the used soil substrate [20], the differences in root metrics cannot be attributed solely to root system plasticity. Destructive measurements at the end of the experiment, or destructive empirical preliminary tests, are still necessary to distinguish whether differences are caused by root plasticity or by measurement-related factors.

The second step of the automated work flow, an algorithm-based root system reconstruction performed on the U-Net segmentation in super-resolution, showed promising results for the MRI datasets. First, the super-resolution segmentation allowed us to derive meaningful automated reconstructions of the MRI*_sand_* data. Since these low CNR data are notoriously difficult to process in automated reconstruction approaches, this in itself is an achievement. Although the recovery rate of the automated tracings is lower than in both manual reconstruction approaches (see Table 3, −5% to M, −17% to M+), root metrics of A are generally on a similar level as M. More intriguingly, differences in the radii derived by M and M+ have a larger impact on root system functioning and simulated root water uptake than the roots missing in A. Hence, we also make substantial errors when processing challenging raw data in our default work flow M. With exception of the root system in Fig. 5C, all geometries derived by the automated work flow for the MRI*_sand_* dataset seem suitable for use in structural-functional plant models (see Fig. 5 and Fig. S5). Same can be stated for the automated reconstructions of the high CNR dataset MRI*_soil_* (see Fig. 6 and Fig. S6). We did not find any statistical differences between the root measures of M, M+, and A (see MRI*_soil_* in Table 3) and also observed a high similarity of the RSAs derived by the 3 reconstruction approaches. Nonetheless, we notice different reconstruction quality of the automated tracings for the 2 MRI datasets. These differences indicate that the performance of the algorithm still depends on the input quality of the segmented images, which was also observed by [46], and is to be expected for automated approaches in general [14]. Although the U-Net segmentation considerably reduces noise and gaps, the gaps and noise remaining in the MRI*_sand_* data still lower the quality of the automated extraction. For the analyzed MRI data, the missing information had a stronger impact on the algorithm than on the human reconstructor, suggesting that automated gap closing remains a challenge even when noise is largely absent. The automated gap closing tends to connect disconnected areas to the nearest neighboring roots, which leads to a substantial inflation of total root tips and derived topology. In contrast, human tracing decisions are based on a broader context of global information when it comes to determining which unconnected root segments are parts of the same root. Local branch orientation is one of the main factors to derive an educated guess during manual reconstruction but is currently not evaluated by the algorithm. Hence, including it as a factor in the automated gap closing procedure could greatly reduce the divergence between M, M+, and A reconstructions caused by differences in the interpretation of missing input data. Nevertheless, the automatic gap closing procedure increases the robustness of the calculated root measures. Although the connectivity is different, most root segments present in the data are recovered.

For both datasets, the topological information derived by the algorithm showed errors resulting from incorrect gap closing (i.e., inflation of root orders) and from general issues related to the logic applied to derive the topology. We suspect that implementing the use of time-series data will allow us to decrease these errors in future iterations of the algorithm. When the automated reconstruction is started with a young root system, fewer roots are present and RLD is usually small. Therefore, it is easier to distinguish between tap/primary roots and the few lateral roots that are present at that growth stage. Gap closing is also less challenging, as the number of potential connection candidates is lower. Subsequently, an MRI scan acquired at a later growth stage can be used to only track root growth that occurred since the initial measurement. By relying on information derived from earlier time points, the complexity of determining topology should be lowered. In addition, the use of time series could also reduce problems related to gaps in the root trajectory. As image quality varies over the course of an experiment, e.g., depending on the irrigation regime and the resulting soil water contents, the visibility of roots and the number of gaps can be expected to fluctuate as well. The use of time-series data could reduce the impact of gaps. Once a root segment is detected, it remains permanently in the reconstruction, whether or not the root signal is present at another measurement time. Such use of time-series data could also help reduce errors associated with merged root structures, which can result from roots coming into direct contact with each other at some point during root growth.

Despite the discussed differences between the reconstructions, the practical benefits of the automated work flow must be evaluated against current common practice. At the moment, data throughput of MRI images is severely hampered by the capabilities of available automated reconstruction methods. Even automated reconstructions derived from MRI images with comparatively high CNR often require manual addition or deletion of certain parts of the root systems (e.g., [20]), or manual determination of topology [58], to retrieve meaningful data. Since we evaluated 2 datasets that reflect the upper and lower boundaries of MRI image quality in terms of CNR and gaps, it is reasonable to assume that the performance of the automated work flow will gradually improve from the quality obtained for the MRI*_sand_* dataset to the quality obtained for the MRI*_soil_* dataset. The automated work flow should therefore enable us to perform MRI experiments in a wider range of soil substrates, as well as at higher soil water contents, since it allows a more efficient use of low CNR data. However, further studies with a wider range of soil substrates are needed to validate this assumption. The results of this work do not necessarily transfer to other plant species. Certain species (i.e., maize), or specific data properties (i.e., missing crown root sections), require specialized adaptations of automated tracing tools to allow meaningful reconstructions. Another example of such complications can be expected for bean root systems. Nodules attached to the root system make automated volume extraction more difficult. It is also to be expected that errors in the second step of the automatic reconstruction process will increase with root system size. Larger root systems tend to be more complex in their architecture, and the containers used in MRI experiments are rather small. As root growth is restricted by the container geometry, RLDs of larger root systems will inevitably increase. The smaller distances between individual roots and an increasing number of roots in direct contact with each other will further complicate automatic volume extraction, gap closing, and the successive derivation of root topology.

At the current state of automated reconstruction methods, visual inspection is essential to ensure qualitative standards are met. In cases of errors that are classified as critical for the intended use of the reconstruction, e.g., using the MRI root system in Fig. 5C for root water uptake simulations, we propose to use the automated reconstruction as scaffold and perform a manual correction. This approach should strike a balance between reconstruction quality and manual effort. Missing and false-positive roots can be easily corrected when using a system like our VR application, and topology can also be corrected.

We found that segmentation via 3D U-Net in super-resolution is a new and beneficial step stone in MRI root reconstruction pipelines that reduces manual reconstruction time, increases root recovery rates, and generally enables automated reconstruction of low-CNR data. In addition, it offers a way to standardize image preprocessing in manual reconstruction work flows, reducing the influence of different human reconstructors on the derived geometries. Hence, the U-Net segmentation should replace simpler segmentation procedures such as global thresholds, which are currently applied in manual and automated reconstruction work flows. For the automated tracing algorithm we could show that U-Net segmentation and super-resolution enables a state-of-the-art performance when deriving tracings for data of high and low CNR. However, topological decision making and gap closing of the tracing algorithm still need further improvements. In future studies, we aim to realize these improvements by factoring in local branch orientation during gap closing and utilizing root order information of different growth stages contained in MRI time-series data. In cases where visual inspection of an automated reconstruction reveals an error that is deemed critical to the intended use, a hybrid work flow would be proposed. Here, the automated reconstruction of the segmented image can be used as a scaffold to which manual corrections are applied in an interactive VR environment. This hybrid work flow should allow us to process larger numbers of root images while maintaining optimal reconstruction quality. It will be investigated by us in further studies.

## Data Availability

CPlantBox is available at https://github.com/Plant-Root-Soil-Interactions-Modelling/CPlantBox, and the automated tracing algorithm is available at https://github.com/JannisHorn/PlantRootExtraction. The VR software for manual reconstruction and the 3D U-Net are available upon request. The data of this study is available from the corresponding author upon request.

## Supplementary Materials

Supplementary 1 Sections S1 to S5 Figs. S1 to S6 Tables S1 to S2 References [59–65]
