# Supplementary material for: 3D U-Net Segmentation Improves Root System Reconstruction from 3D MRI Images in Automated and Manual Virtual Reality Work Flows
Source: Plant Phenomics. 2023 Jul 28;5:0076. doi: 10.34133/plantphenomics.0076 (PMC10381537; doi:10.34133/plantphenomics.0076)
Supplement: Supplementary 1 — Sections S1 to S5 Figs. S1 to S6 Tables S1 to S2 References [59–65] [file plantphenomics.0076.f1.pdf]

Supplemental Material

S1 MRI experiment and image processing

S1.1 Experimental description

The MRI scans used in this work were gathered in an experiment carried out at the Forschungszentrum Juelich. Eight seeds of white lupine *(Lupinus albus)* were germinated on wet paper for four days and planted into two different soil substrates: four seedlings were planted into PVC cylinders filled with a sandy loam and four seedlings were planted into cylinders containing natural sand. The sandy loam was taken from the topsoil of an agricultural field close to Kaldenkirchen, Germany, with a texture of 73 % sand, 23 % silt and 4 % clay, a soil organic carbon content of 0.85 g 100 g^{-1} and a small fraction (< 0.0025 g g^{-1}) of ferromagnetic particles [**Pohlmeier2009**]. It was dried and sieved to 2 mm, and is denoted as "soil". The natural sand had a mean grain size of approximately 0.3 mm (FH31, Quarzwerke Frechen GmbH, Frechen, Germany) and is denoted as "sand". Used cylinders were 21 cm in height, had an inner diameter of 5.6 cm, and were filled with the respective soil substrate up to a height of 18.5 cm. Additionally, a marker tube filled with 80:20 D_2O/H_2O (w/w) was inserted into each cylinder, which was used later to normalize voxel intensities of the MRI images. To prevent evaporation, a layer of coarse gravel was used as a capillary barrier at the soil surface. The cylinders were perforated at the bottom and the holes were covered with a nylon mesh. Subsequently, the substrate-filled cylinders were saturated from the bottom to saturation soil water contents of 0.36 cm^3 cm^{-3} for soil and 0.38 cm^3 cm^{-3} for sand. Water contents were measured gravimetrically and converted to volumetric water contents via the soil volume. Bulk densities ranged between 1.45 and 1.49 g cm^{-3} for sand, and 1.39 and 1.42 g cm^{-3} for soil. Plants were grown for 8 to 15 days in a laboratory at a relative humidity of approximately 45 %, a temperature of approximately 25°C and a day-night cycle of 12 h/12 h. Photosynthetic active radiation (PAR) during the day was 450 ± 50 μmol m^{-2} s^{-1}. Irrigation was performed with tap water, consistently every second to third day from the top with a syringe to compensate for half of the transpiration loss. For the plants grown in sand, the tap water was additionally mixed with half strength Hoagland nutrient solution. The experimental containers were scanned by MRI at different time points (Tab. 1). Subsequently, the roots were excavated and washed. They were then scanned with an Epson flatbed scanner with a resolution of 0.005 mm in horizontal and 0.01 mm in vertical direction. The scans were analyzed with WinRHIZO (Regent Instruments, Ottawa, Canada) to determine total root length.

S1.2 MRI image processing

All pre-analysis image-processing steps of MRI scans were performed using MATLAB 2015a (The MathWorks Inc., Cambridge, UK). Due to the gradient non-linearity artefact in our MRI system, the raw MRI scans showed systematic distortions increasing from the center line in both vertical directions [**Heinrichs1992**]. We built a calibration phantom using the same PVC cylinders as in the experiment with soil-grown lupine roots, which was filled with sand as well as four marker tubes that were filled with 80:20 D_2O / H_2O (w/w). The phantom was then scanned using the same setup

(sequence, resolution). The undistorted center slice of the scanned MRI phantom stack was used as reference slice. Using affine transformation, each horizontal slice up and down the center slice was registered to the reference slice and dewarped. The generated transformation matrices were subsequently used to de-warp the MRI lupine scans slice-by-slice. As mentioned above, each column was scanned in three sections with an overlap between 5 and 10 mm. These scans were subsequently stitched together using the following approach: first, we normalized the voxel intensities of the different sections using the intensities of the marker tube as a reference. Next, we determined the two overlapping slices of two neighboring sections using the criteria of maximal correlation. The overlapping slice of the lower section was then mapped to the overlapping slice of the upper section using an affine transformation. All remaining slices of the lower section were then equally registered with the slice above using the same transformation matrix. Finally, the three sections were concatenated. Subsequently, the connected structure of the marker tube was masked and excluded from the MRI scans. The images pre-processed in this way were the starting point for the three root reconstruction work flows examined in this paper (Fig. 1).

S2 Modifications to automated reconstruction algorithm

S2.1 Modification to Dijkstra's shortest path algorithm

To allow a more complete extraction of roots from imperfect data (i.e. data with gaps), Dijkstra's shortest path algorithm [**Dijkstra1959**] is modified. The path-cost threshold is momentarily ignored to allow the exploration of high-cost voxels within the maximum gap length. Allowing such traversals requires a new cost-map, as using the initial cost-map (Fig. 3b) would result in the emergence of multiple high-cost paths from disconnected segments of the same root. Hence, the initial cost-map is adapted when using the gap closing option: voxels below the given minimum intensity threshold are not excluded from the cost map, but their cost is increased by a factor of ten (Fig. 3c). Subsequently, all voxels above half of the maximum voxel cost are considered as potential gaps. This preserves low-intensity information while simultaneously enhancing the contrast between gap and no-gap voxels. Penalizing these low-intensity voxels also ensures that the shortest path algorithm explores no-gap positions before considering gap positions. When the shortest path algorithm arrives at a no-gap position, where all adjacent voxels are classified as gap positions, it explores all voxels within the predefined maximum gap length. If a voxel classified as no-gap position can be found within this perimeter, it is assumed that the gap positions between the two no-gap positions is caused by missing root information and the gap is bridged. The cost of the path that traverses the gap is adjusted, because it is assumed that it should have crossed a missing lower-cost area. Since the adjusted path cost is now below the path-cost threshold, the gap separating the root segments is closed and exploration continues at the found no-gap position on the other side of the gap.

S2.2 Modification to the 3D curve skeletonization algorithm

In the second stage of the automated reconstruction algorithm a modified version of the 3D curve skeletonization algorithm described in Jin et al. [**Jin2016**] is used to extract a root structure graph.

A radius estimate map is extracted from the largest connected component (Fig. 3d). This is used as basis for another cost map used for the Dijkstra's shortest path algorithm [**Dijkstra1959**]. Larger radius estimates result in lower traversal cost. Local maxima in the radius-estimate map are extracted. These quench-points are used as potential root tips and sorted by their respective distance to the selected shoot position, starting with the farthest point. As long as unused quench points are available, a connection is made between the shoot and the topmost quench point in the list. This connection is made along the shortest path, according to the radius-based cost map. The resulting connection follows the path of highest radius estimate and should therefore follow the center of the segmented root. Quench points in the vicinity of this extracted path are identified based on an enlarged radius search along the path and deleted from the list. Not starting connections in a tube around paths reduces the number of wrong subbranches from noisy data. If an already extracted branch is reached, the current extraction is connected to the existing root graph. The resulting root graph contains a node for each connected voxel. To reduce the number of nodes, the Douglas-Peucker algorithm [**Douglas1973**] is used. Radius estimates for each node are taken from the generated radius map. Root parameters are computed based on this radius estimate and the voxel size of the data.

S3 Calculation of quantitative measures

RL (cm) denotes the total root length of a root system. The root length density, RLD (cm cm^{-3}), is calculated based on the container volume V_c:

$$\mathrm{RLD} = \frac{\mathrm{RL}}{V_\mathrm{c}}. \tag{S1}$$

For the MRI experiment, V_c was $455\,\mathrm{cm}^3$. The half-mean-distance between roots, HMD (cm), is also based on the root length per container volume and approximated following the classical approach proposed by Newman [**Newman**]:

$$\mathrm{HMD} = (\pi \times \mathrm{RLD})^{-\frac{1}{2}}. \tag{S2}$$

We calculate the mean radius of a root system as

$$r_\mathrm{mean} = \sum_{i=0}^{n} \frac{l_i}{\mathrm{RL}} r_i, \tag{S3}$$

where r_i is the radius of a root segment, l_i is the length of a root segment, and n is the total number of root segments of a given root system.

The total number of root tips per tracing, as well as the number of lateral root tips is given as a topology measure. Here, we specify lateral tips up to the highest order observed in the manual reconstructions (3rd order lateral roots). Although lateral roots above 3rd order are recorded in the automatic tracings, we refrain from stating them in the root measures results section for improved readability. Laterals above 3rd order are qualitatively accessible in the visual comparisons.

We compute the equivalent conductance of the root system, K_{rs} (cm^2 d^{-1}), according to Meunier et al. [**MEUNIER2017**] as

$$K_{\mathrm{rs}} = \frac{T_{\mathrm{act}}}{\Psi_{\mathrm{sr}} - \Psi_{\mathrm{collar}}}, \tag{S4}$$

where T_{act} (cm^3 day^{-1}) is the actual transpiration rate, Ψ_{sr} (cm) is the mean soil water potential at the soil-root interfaces, and Ψ_{collar} (cm) is the water potential at the root collar. We chose a scenario where Ψ_{collar} is set to -15000 cm, applied as Dirichlet boundary condition, and Ψ_{sr} is set to -500 cm at the soil surface, while assuming a hydrostatic equilibrium in the soil domain. K_{rs} reflects the ability of a root system to take up a certain water volume under a given water potential difference between root collar and soil. T_{act} results from solving water flow in the roots according to Meunier et al. [**MEUNIER2017**]. We calculate the root xylem potential of each root segment and use it to derive the respective radial volumetric flow (cm^3 day^{-1}). Finally, summation of the radial flows yields T_{act}.

The standard uptake fraction of a root segment, SUF_i (-), indicates its relative contribution to T_{act} and is calculated via

$$\mathrm{SUF}_i = \frac{J_{\mathrm{r}}}{T_{\mathrm{act}}}, \tag{S5}$$

where J_{r} is the radial water flux into a root segment (cm^3 day^{-1}). To obtain an aggregated (scalar) metric, we determine the mean depth of standard root water uptake, zSUF (cm), by

$$\mathrm{zSUF} = \sum_{i=0}^{n} z_i \times \mathrm{SUF}_i, \tag{S6}$$

where z_i (cm) is the respective depth of a root segment.

Finally, we investigate in how far the root hydraulic properties affect the impact of differences in tracings of the three reconstruction methods on root system function metrics. In the first scenario, called constant scenario, we calculate the metrics by applying the same fixed axial and radial conductivities to all roots. Based on Zarebanadkouki et al. [**Zarebanadkouki**], we apply a fixed axial conductivity of k_{z}= 4.32e^{-2} (cm^3 d^{-1}) and a fixed radial conductivity of k_{r} = 1.73e^{-4} d^{-1} to all roots. For the second scenario, called variable scenario, we apply order and age-dependent root hydraulic properties. As we did not reconstruct time-series, we linearly interpolate root segment ages as a function of root length while assuming a daily growth rate of 1 cm. A resulting age distribution for an exemplary 14-day old root system is shown in Fig. S1. Parameterized conductivity values are again based on Zarebanadkouki et al. [**Zarebanadkouki**] and depicted in Fig. S2. We compute the functional metrics for the constant scenario (K_{rsc}, $\mathrm{zSUF_c}$) as well as for the variable scenario (K_{rsv}, $\mathrm{zSUF_v}$).

Three additional measures, not directly related to the RSAs, are included in the results section to enable a more in-depth classification of the results.The available root length information from WinRHIZO measurements, $\mathrm{RL_{WR}}$, is used to calculate the recovery rate, RR (%), of a respective tracing:

$$\mathrm{RR} = \frac{\mathrm{RL}}{\mathrm{RL_{WR}}} \times 100. \tag{S7}$$

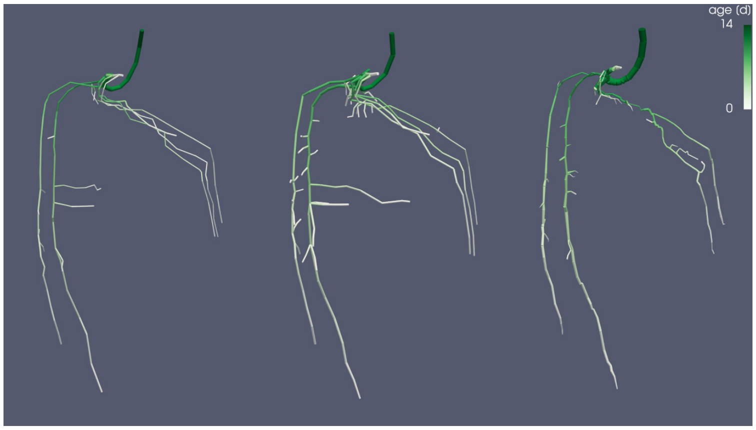

Figure S1: Age-distribution used to assign age-dependent root hydraulic conductivities to manual tracings M (left), manual tracings based on segmented images M+ (middle), and automated tracings A (right). Shown is an exemplary 14-day old *Lupinus albus* root system of the MRI dataset.

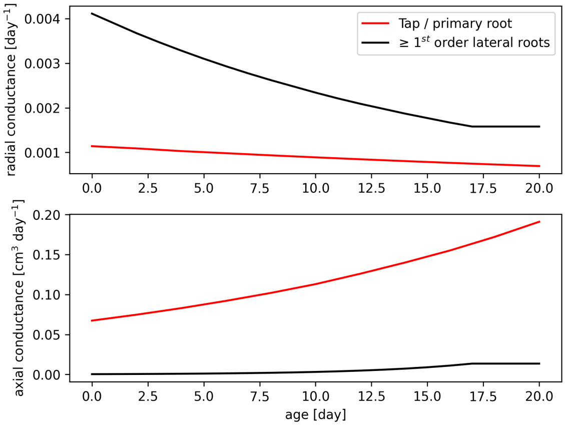

Figure S2: Age-dependent root hydraulic conductivities applied in the variable simulation scenario.

For the M and M+ reconstructions, we also report the respective reconstruction speed, v_r (cm root min^{-1}), which is calculated as

$$v_\mathrm{r} = \frac{t_\mathrm{r}}{\mathrm{RL}}, \tag{S8}$$

where t_r (min) is the total time required to trace a root system. Division by the respective RL is done to mitigate differences in total root length on t_a between M and M+ reconstructions.

To give an estimate of the quality of the MRI images, we calculate an exemplary contrast-to-noise-ratio, CNR (-), for the raw images. The raw data is loaded into Fiji [**Fiji**] and an image slice roughly located at medium depth of the experimental containers (-10 cm) is used to manually place a circular selection around the area of the layer with highest, homogeneous signal intensity I_s (-), i.e. the root center of the thickest root. A second circular selection is placed around a soil region containing background noise of intensity I_b, i.e. the largest possible area of the soil cylinder slice

that does not contain roots. The mean intensities of the two areas are used to calculate the CNR as

$$\mathrm{CNR} = \frac{I_\mathrm{s} - I_\mathrm{b}}{\sigma_{I_\mathrm{b}}}, \tag{S9}$$

where σ_{I_b} is the standard deviation of I_b.

S4 Complete root system reconstructions

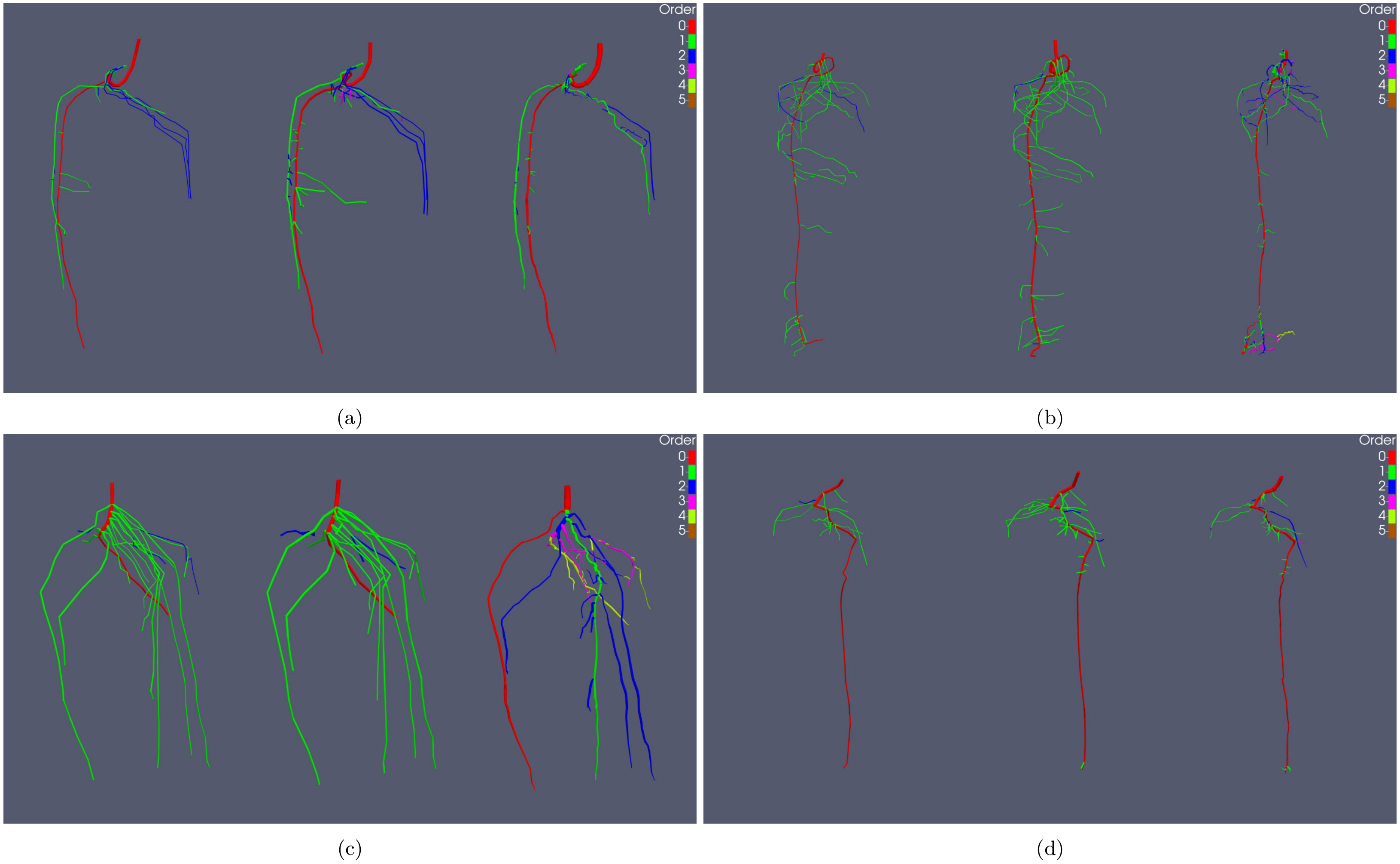

(a) (b) (c) (d)

Figure S3: Manual tracings M (left), manual tracings after segmentation M+ (middle) and automated tracings A (right) of four *Lupinus albus* root systems (a-d) grown in sand derived by MRI scans. Colors display root orders, root segments are scaled by their respective radius. Age of the root systems is between 8 and 14 days (see Tab. 1).

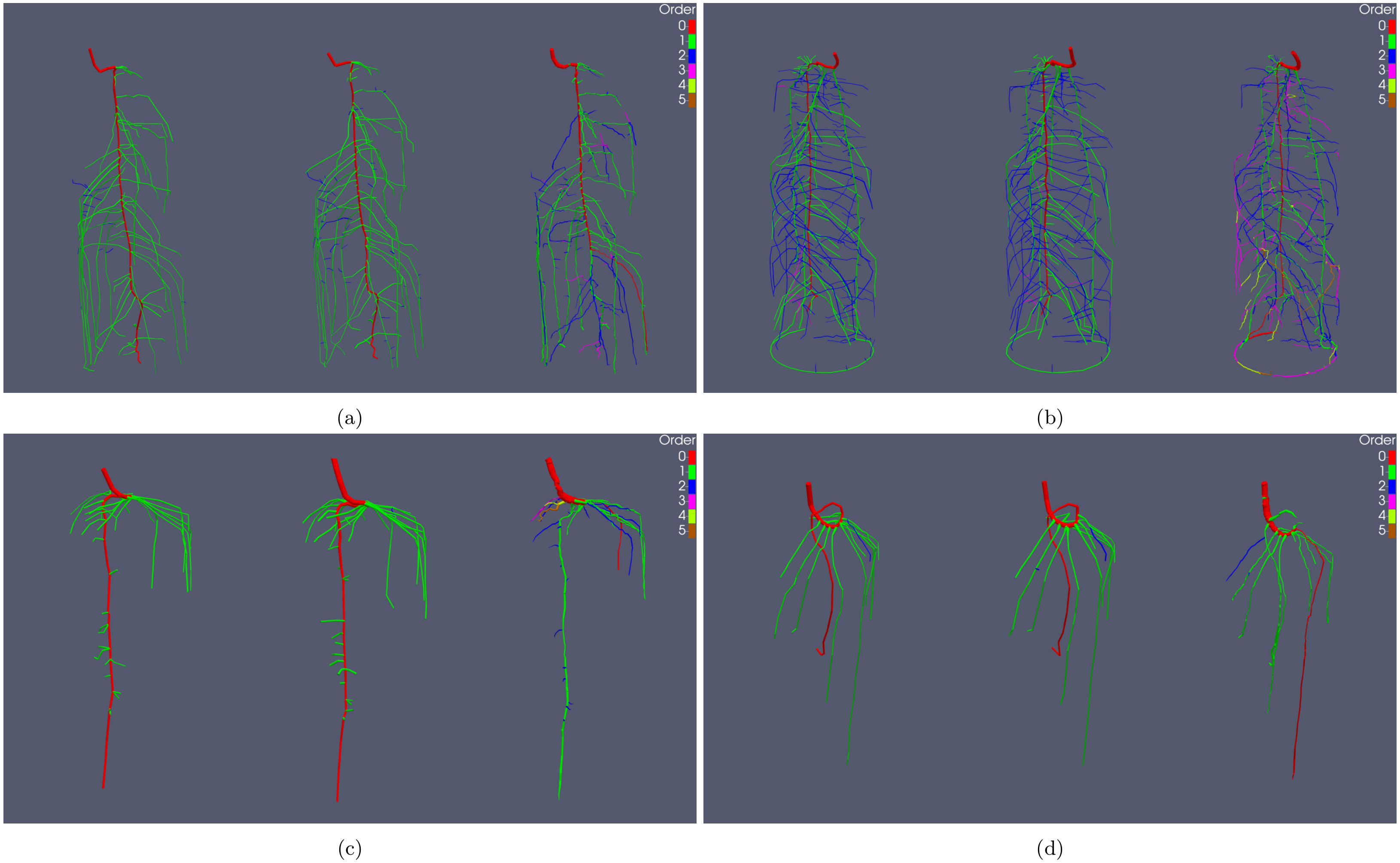

Figure S4: Manual tracings M (left), manual tracings after segmentation M+ (middle) and automated tracings A (right) of four *Lupinus albus* root systems (a-d) grown in soil derived by MRI scans. Colors display root orders, root segments are scaled by their respective radius. Age of the root systems is between 8 and 15 days (see Tab. 1).

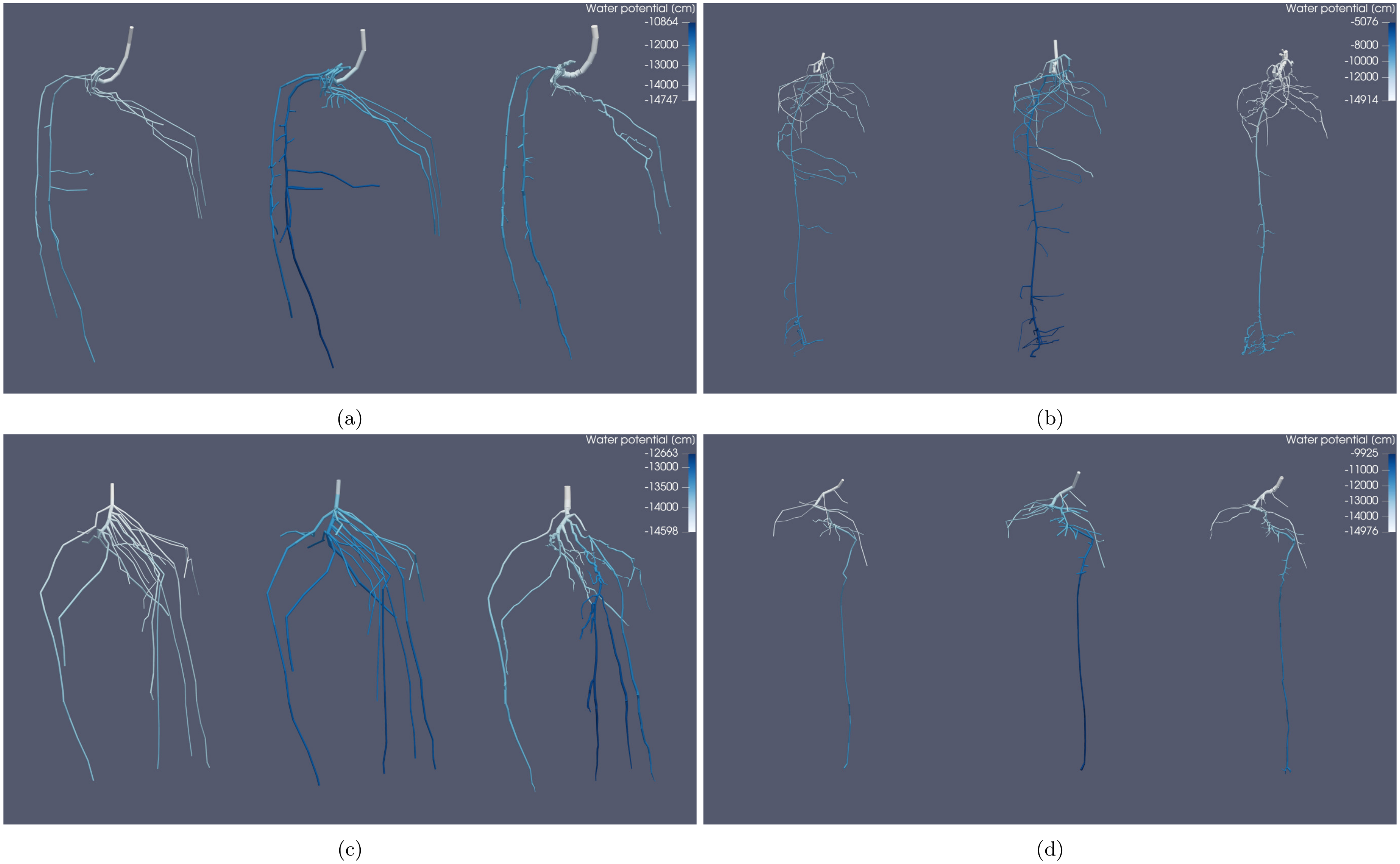

Figure S5: Manual tracings M (left), manual tracings after segmentation M+ (middle), and automated tracings A (right) of four *Lupinus albus* root systems (a-d) grown in sand derived by MRI scans. Age of the root systems is between 8 and 14 days (see Tab. 1). Shown is the root water potential (cm) of the constant simulation scenario, scaled to the maximal and minimal potentials observed for the three root systems shown in each subfigure. Root segments are scaled by their respective radius.

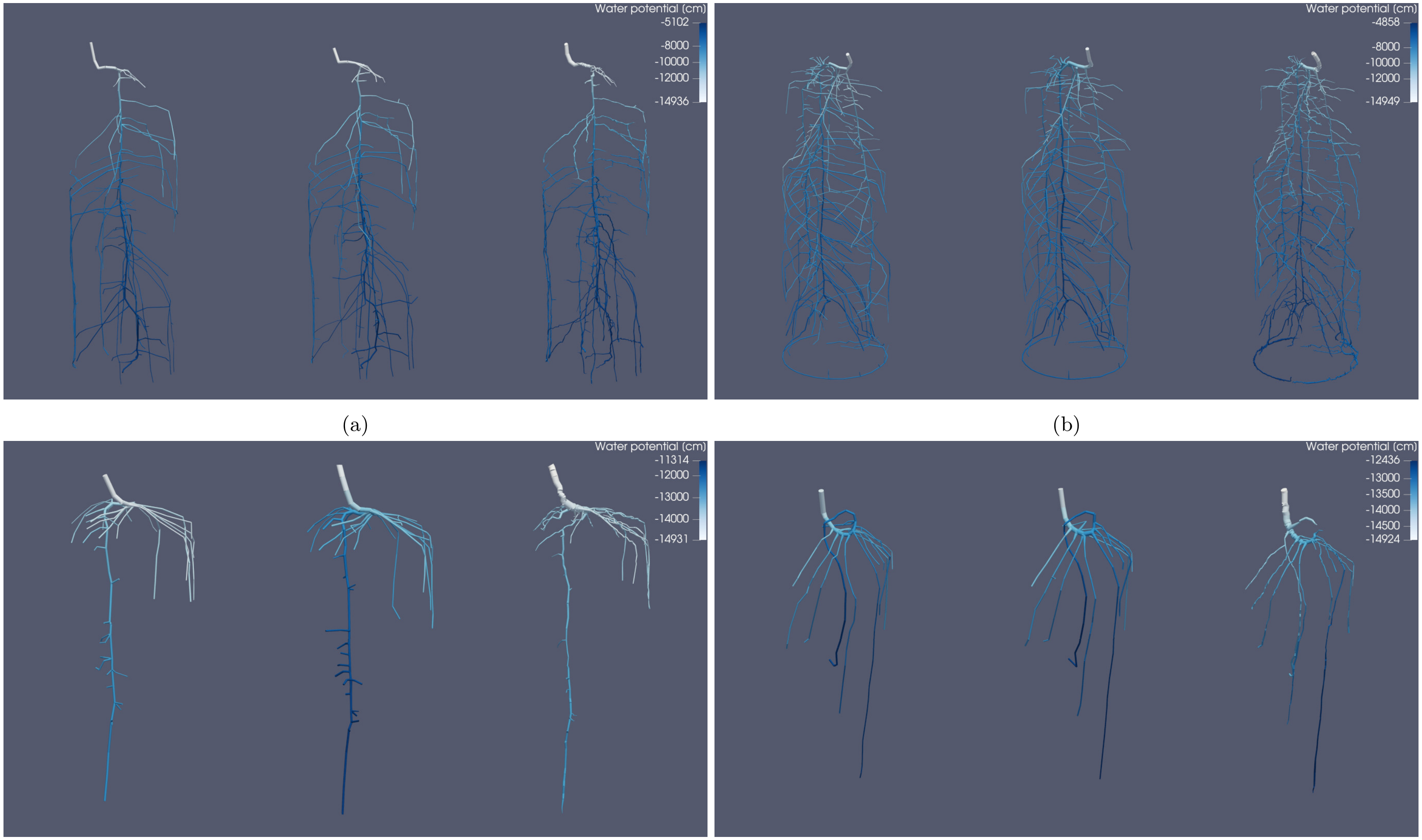

(a) (b)

(c) (d)

Figure S6: Manual tracings M (left), manual tracings after segmentation M+ (middle), and automated tracings A (right) of four *Lupinus albus* root systems (a-d) grown in soil derived by MRI scans. Age of the root systems is between 8 and 15 days (see Tab. 1). Shown is the root water potential (cm) of the constant simulation scenario, scaled to the maximal and minimal potentials observed for the three root systems shown in each subfigure. Root segments are scaled by their respective radius.

S5 Comparison of root measures on single-plant basis

Table S1: Comparison of root measures for *Lupinus albus* tracings derived by MRI scans. MRI_{sand} is comprised of the four root systems grown in sand. M denotes manual tracings derived using unaltered MRI images, M+ denotes manual tracings performed on the U-Net segmentations, A denotes tracings derived by the two-step automated work flow. Descriptions of the quantitative measures are given in Section 2.4.2 , equations of measures and descriptions of the constant and variable simulation scenarios are given in supplemental material S3. Note that K_{rs} and zSUF are simulated and not measured quantities (see Eq. S4 - Eq. S6).

**Dataset**	$\mathbf{MRI}_{sand}$											
# of root system	1			2			3			4		
RL_{WR} [cm]	137			233			105			97		
Root system age [d]	14			14			8			8		
Reconstruction method	M	M+	A	M	M+	A	M	M+	A	M	M+	A
CNR [-]	15.78	-	-	9.42	-	-	11.38	-	-	7.57	-	-
RL [cm]	64.57	84.09	56.29	148.28	185.73	138.63	98.23	97.46	84.68	55.93	74.13	58.82
Recovery rate [%]	47.13	61.38	41.09	63.64	79.81	59.50	93.55	92.82	80.65	57.66	76.42	60.42
RLD [cm cm^{-3}]	0.14	0.18	0.12	0.33	0.41	0.30	0.22	0.21	0.19	0.12	0.16	0.13
HMD [cm]	1.50	1.31	1.60	0.99	0.88	1.02	1.21	1.22	1.31	1.61	1.40	1.57
r_{mean} [mm]	0.248	0.329	0.359	0.232	0.298	0.280	0.251	0.336	0.318	0.305	0.391	0.337
# of roots [-]	17	38	33	37	55	111	18	19	36	15	32	31
# of 1st laterals [-]	8	17	13	32	50	35	14	14	12	13	29	21
# of 2nd laterals [-]	8	15	16	4	4	40	3	4	9	1	2	6
# of 3rd laterals [-]	0	5	3	0	0	26	0	0	12	0	0	3
K_{rsc} [cm^2 day^{-1}]	1.52E-03	2.38E-03	1.87E-03	2.51E-03	3.20E-03	3.19E-03	2.49E-03	3.11E-03	2.60E-03	1.62E-03	2.50E-03	1.83E-03
K_{rsv} [cm^2 day^{-1}]	7.28E-03	1.07E-02	9.04E-03	1.20E-02	1.39E-02	1.71E-02	1.11E-02	1.21E-02	5.61E-03	9.50E-03	1.36E-02	1.01E-02
$zSUF_c$ [cm]	-4.05	-4.16	-4.17	-5.74	-5.40	-5.80	-3.51	-3.39	-3.76	-4.88	-4.08	-4.74
$zSUF_v$ [cm]	-3.48	-3.48	-3.31	-4.68	-3.78	-4.25	-1.95	-1.83	-2.71	-3.91	-3.13	-3.90
v_r [cm min^{-1}]	2.21	4.86	-	2.52	5.96	-	4.51	8.03	-	3.91	7.12	-

Table S2: Comparison of root measures for *Lupinus albus* tracings derived by MRI scans. MRI_{soil} is comprised of the four root systems grown in Kaldenkirchen soil. M denotes manual tracings derived using unaltered MRI images, M+ denotes manual tracings performed on the U-Net segmentations, A denotes tracings derived by the two-step automated work flow. Descriptions of the quantitative measures are given in Section 2.4.2, equations of measures and descriptions of the constant and variable simulation scenarios are given in supplemental material S3. Note that K_{rs} and zSUF are simulated and not measured quantities (see Eq. S4 - Eq. S6).

**Dataset**	**MRI_{soil}**											
# of root system	5			6			7			8		
RL_{WR} [cm]	323			508			85			84		
Root system age [d]	14			15			9			8		
Reconstruction method	M	M+	A	M	M+	A	M	M+	A	M	M+	A
CNR [-]	133.26	-	-	132.58	-	-	160.80	-	-	257.54	-	-
RL [cm]	269.24	293.15	276.57	488.34	480.69	473.02	68.56	76.02	64.37	76.77	75.61	68.02
Recovery rate [%]	83.36	90.76	85.63	96.13	94.42	93.11	80.66	89.44	75.73	91.39	90.01	80.89
RLD [cm cm^{-3}]	0.59	0.64	0.61	1.07	1.06	1.04	0.15	0.17	0.14	0.17	0.17	0.15
HMD [cm]	0.73	0.70	0.72	0.54	0.55	0.55	1.45	1.38	1.50	1.37	1.38	1.46
r_{mean} [mm]	0.231	0.228	0.243	0.223	0.230	0.220	0.288	0.332	0.284	0.305	0.337	0.312
# of roots [-]	75	109	124	192	199	267	35	39	34	15	15	17
# of 1st laterals [-]	60	59	37	45	44	39	34	37	11	13	12	13
# of 2nd laterals [-]	14	49	64	137	146	127	0	1	18	1	2	3
# of 3rd laterals [-]	0	0	20	9	8	73	0	0	1	0	0	0
K_{rsc} [cm^2 day^{-1}]	3.18E-03	3.42E-03	3.30E-03	6.03E-03	5.70E-03	5.50E-03	1.90E-03	2.28E-03	1.77E-03	2.23E-03	2.42E-03	2.05E-03
K_{rsv} [cm^2 day^{-1}]	1.31E-02	1.42E-02	1.25E-02	1.63E-02	1.61E-02	1.44E-02	1.33E-02	1.39E-02	8.65E-03	1.09E-02	1.15E-02	1.04E-02
$zSUF_c$ [cm]	-9.28	-9.04	-9.36	-9.73	-9.53	-9.75	-3.35	-3.26	-3.08	-3.47	-3.46	-3.62
$zSUF_v$ [cm]	-6.64	-6.30	-6.04	-5.38	-5.02	-5.81	-2.96	-2.92	-1.75	-2.17	-2.16	-2.59
v_r [cm min^{-1}]	7.67	9.37	-	6.19	8.61	-	4.29	5.05	-	5.02	6.51	-
